# Effects of the Licorice Isoflavans Licoricidin and Glabridin on the Growth, Adherence Properties, and Acid Production of *Streptococcus mutans*, and Assessment of Their Biocompatibility

**DOI:** 10.3390/antibiotics10020163

**Published:** 2021-02-05

**Authors:** Katy Vaillancourt, Geneviève LeBel, Geneviève Pellerin, Amel Ben Lagha, Daniel Grenier

**Affiliations:** Oral Ecology Research Group, Faculty of Dentistry, Université Laval, Québec City, QC G1V 0A6, Canada; katy.vaillancourt@greb.ulaval.ca (K.V.); genevieve.lebel.4@ulaval.ca (G.L.); genevieve.pellerin.3@ulaval.ca (G.P.); amel.ben-lagha.1@ulaval.ca (A.B.L.)

**Keywords:** adherence, biofilm, cariogenic bacteria, dental caries, glabridin, licorice, licoricidin, *Streptococcus mutans*

## Abstract

Pharmacological studies have linked a number of human health benefits with licorice due to its anticancer, anti-inflammatory, anti-oxidant, and antimicrobial properties. The aim of this study was to investigate the effects of licoricidin and glabridin, two major licorice isoflavans, on growth and virulence properties (biofilm formation, acid production, dextran production, adherence) of the cariogenic bacterium *Streptococcus mutans*. Moreover, the biocompatibility of these licorice compounds was assessed in an in vitro model of oral keratinocytes. We used a broth microdilution assay to show that licoricidin and glabridin exhibit a marked antibacterial activity against *S. mutans*. Glabridin and, to a lesser extent, licoricidin reduced the biofilm viability of *S. mutans*. In addition, glabridin decreased the production of dextran by *S. mutans*. The two licorice isoflavans attenuated the adherence of *S. mutans* to a saliva-coated hydroxylapatite surface, and reduced acid production from glucose. Lastly, depending on the concentrations tested, the two licorice isoflavans showed no or low toxicity toward oral keratinocytes. Within the limitations of this study, our data suggest that licoricidin and glabridin may be promising agents for controlling dental caries.

## 1. Introduction

Dental caries is a chronic infectious disease associated with the progressive destruction of the hard tooth structures (enamel, dentin, and cementum) resulting from the metabolism of acidogenic/aciduric bacteria embedded in dental biofilms. This disease affects over 35% of people of every age group worldwide, particularly in developing countries [1]. *Streptococcus mutans* is considered the most important cariogenic bacterium [2]. This Gram-positive bacterium metabolizes exogenous dietary carbohydrates and produces organic acids, mainly lactic acid, that drives the dissolution of calcium and phosphate in the hydroxylapatite crystal structure of teeth [2]. *S. mutans* glycosyltransferases produce extracellular polysaccharides that contribute to the formation of a dense and adherent biofilm, which retains acids on the tooth surface and increases resistance to antimicrobial agents [3].

Dental caries can be prevented by regular tooth brushing and flossing. However, efficient removal of dental plaque is not always possible, especially by handicapped or elderly individuals who lack the necessary dexterity or motivation. To overcome these shortcomings, mouthwashes supplemented with chemoprophylactic agents that inhibit the growth, adhesion, and biofilm formation of cariogenic bacteria can be used as adjuncts to good oral hygiene practices [4,5]. As a number of undesirable effects, including tooth staining, unpleasant taste, and the emergence of bacterial resistance, have been associated with the chemoprophylactic agents added to mouthwashes [6,7], there is a need to identify alternatives.

In recent years, there has been growing interest in studying plant-derived molecules as effective and safe substances for the management of dental caries [8,9]. Licorice is the root of *Glycyrrhiza uralensis* Fisch., *Glycyrrhiza glabra* L., and *Glycyrrhiza inflata* Bat. and has been used for thousands of years as a traditional herbal remedy in China and Far Eastern countries for treating gastrointestinal disorders, chronic hepatitis, rheumatoid arthritis, and many other diseases [10,11]. Licorice and its constituents are Generally Recognized as Safe (GRAS) for use in foods and over-the-counter drugs by the United States Food and Drug Administration (FDA) (21 CFR 184.1408; 310.528; 310.544; 310.545) [12]. To date, more than 300 flavonoids have been isolated from licorice, including flavones, flavonols, flavanols, isoflavones, and isoflavans [13]. Pharmacological studies have associated several human health benefits with licorice due to its anticancer, anti-inflammatory, anti-oxidant, and antimicrobial properties [13,14,15]. Licorice compounds may also be beneficial for treating oral health conditions [16]. Although the anticariogenic properties of licorice have been touted for over 30 years, few studies on this aspect have been published. A licorice root extract (*G. uralensis*), which is known to kill *S. mutans* [17], has been incorporated into lollipops in order to reduce dental caries in children. These licorice lollipops have shown promise in reducing dental caries by decreasing *S. mutans* levels in saliva [18]. Glycyrrhizin, a sweet-tasting compound (50 times sweeter than sucrose), is the main triterpenoid saponin glycoside of *G. glabra* root and has been the subject of several investigations. Segal et al. [19] showed that while the growth of *S. mutans* is not affected by glycyrrhizin in the presence of sucrose, its ability to adhere to a glass surface was almost completely inhibited. Sela et al. [20] showed that glycyrrhizin dose-dependently inhibits the glucosyltransferase activity of *S. mutans*, which is involved in the formation of water-insoluble glucans required for biofilm formation. The aim of this study was to investigate the effects of licoricidin and glabridin, two major isoflavans of licorice, on the growth and virulence properties (biofilm formation, acid production, dextran production, and adherence) of *S. mutans*. The biocompatibility of these licorice compounds was also assessed using an oral keratinocyte model.

## 2. Results and Discussion

**Figure 1:**
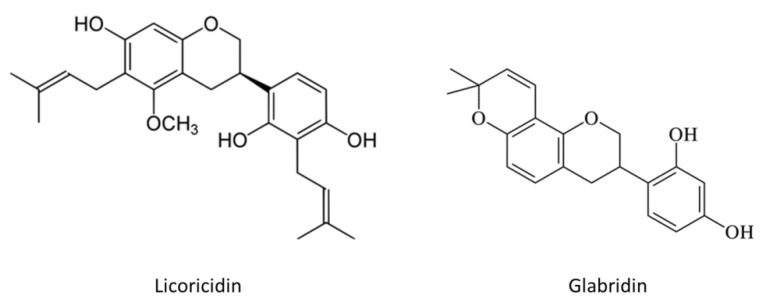
Chemical structures of licoricidin and glabridin.

Licoricidin and glabridin are major isoflavans found in licorice and are characterized by the presence of a 3-phenylchromen backbone (Figure 1). The antibacterial activities of the two licorice compounds were investigated by determining their minimal inhibitory concentrations (MICs) and minimal bactericidal concentrations (MBCs) against one reference (ATCC 25175) and four clinical (12A, 33A, INB, T8) strains of *S. mutans* in a broth microdilution assay (Table 1). On the one hand, licoricidin had a MIC of 6.25 µg/mL and a MBC varying between 6.25 and 25 µg/mL. On the other hand, glabridin showed a MIC in the range of 6.25 to 12.5 µg/mL and a MBC varying between 6.25 and 25 µg/mL. In previous studies, licoricidin has been reported to exert antibacterial activity against *Porphyromonas gingivalis* and *Prevotella intermedia,* two periodontal pathogens [21] as well as against the endodontic pathogen *Enterococcus faecalis* [22]. Although we did not investigate the mechanism of antibacterial action of the isoflavans tested, a previous study by Araya-Cloutier et al. [23] showed that glabridin causes membrane permeabilization as determined by the uptake of the fluorescent probe propidium iodide. Other flavonoids isolated from licorice root, including 1-methoxyficifolinol, 6,8-diprenylgenistein, 6,8-diisoprenyl-5,7,4′-trihydroxyflavone, gancaonin G, glycyrrhizol A, glycyrrhizol B, and licorisoflavan A have also been reported to exhibit antibacterial activity against *S. mutans* [17,24].

Mature bacterial biofilms are usually more resistant to antibacterial agents than planktonic bacteria. We determined the effect of a 2-h treatment with licoricidin and glabridin (at their MIC and 2× MIC) on the viability of *S. mutans* biofilms using a Filmtracer™ Live/Dead Biofilm Viability kit. A treatment with glabridin at 2× MIC significantly (*p* < 0.001) reduced the biofilm viability for four of the five strains of *S. mutans* tested (Table 2). Licoricidin, even at 2× MIC, was less effective than glabridin. Neither licoricidin nor licoricidin caused a detachment of the preformed *S. mutans* biofilms as determined by crystal violet staining (data not shown).

**Figure 2:**
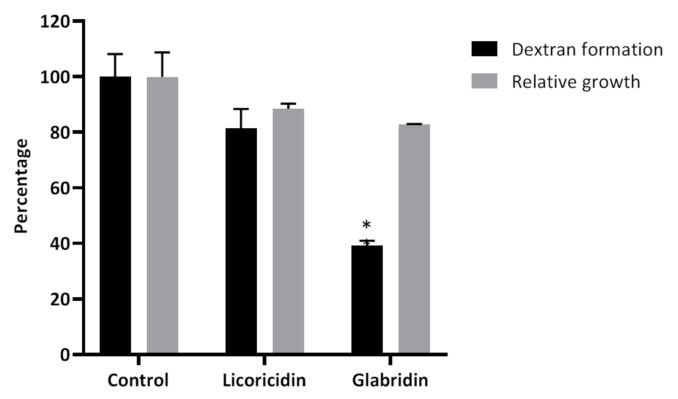
Effects of licoricidin and glabridin (¼ MIC) on dextran formation by and the growth of *Streptococcus mutans* ATCC 25175. Dextran formation was determined using Cascade Blue-conjugated dextran dye while growth was assessed by monitoring the A_660_. The assays were carried out in triplicate, and the means ± standard deviations were calculated. *, significantly different (*p* < 0.001) from the control (no licorice compounds).

Dextran is formed by *S. mutans* from exogenous sucrose and is crucial for the establishment of cariogenic biofilms [3]. Figure 2 shows that glabridin, but not licoricidin, significantly (*p* < 0.001) decreased the production of dextran by *S. mutans* ATCC 25175. The decrease was not associated with a significant reduction in bacterial growth.

**Figure 3:**
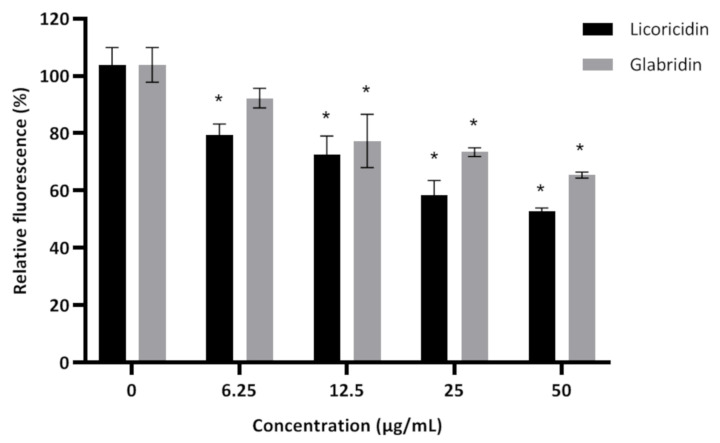
Effect of licoricidin and glabridin on the adherence of fluorescein isothiocyanate (FITC)-labeled *Streptococcus mutans* ATCC 25175 cells to a saliva-coated hydroxylapatite surface. The number of adhered bacteria was assessed by determining the relative fluorescence units (excitation wavelength 495 nm; emission wavelength 525 nm). The assays were carried out in triplicate, and the means ± standard deviations were calculated. *, significantly different (*p* < 0.001) from the control (no licorice compounds).

The formation of dental biofilms is initiated by the adhesion of planktonic bacteria to tooth enamel. We used a saliva-coated hydroxylapatite surface model to determine whether licoricidin and/or glabridin can impair the adhesion of *S. mutans* ATCC 25175. As shown in Figure 3, both glabridin and licoricidin dose-dependently and significantly (*p* < 0.001) reduced bacterial adherence to saliva-coated hydroxylapatite. More specifically, at a concentration of 25 µg/mL, licoricidin reduced the adherence of *S. mutans* by 41.6%, while glabridin reduced the adherence of *S. mutans* by 26.6%.

**Figure 4:**
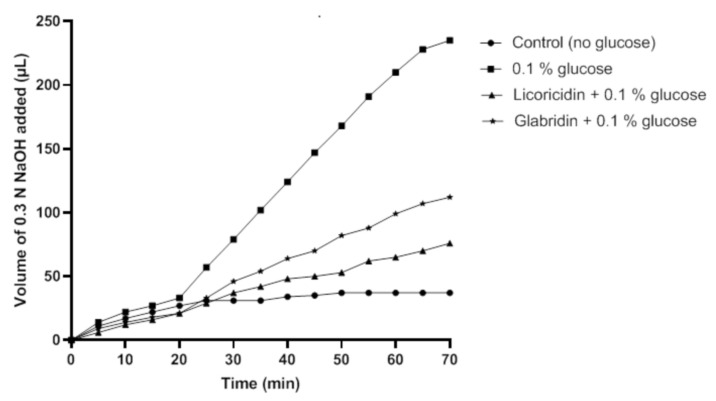
Effect of licoricidin and glabridin on acid production by *Streptococcus mutans* ATCC 25175. Glycolysis was monitored at a constant pH (7.0) by automatic titration of the acid produced. The volume of 0.3 N NaOH required to maintain a constant pH of 7.0 was determined over a 70-min period at 37 °C. Four independent experiments were performed, and a representative set of data is presented.

Then, we investigated the effect of licoricidin and glabridin, at a concentration equal to 1× MIC for which these licorice compounds had no bactericidal effect, on the glycolytic pH decrease caused by *S. mutans* incubated in the presence of glucose. As shown in Figure 4, in the absence of the licorice compounds, 226.7 µL of 0.3 N NaOH were necessary to maintain a neutral pH of the bacterial suspension following a 70-min incubation. The presence of either licoricidin or glabridin reduced the amount of 0.3 N NaOH required to maintain a neutral pH, indicating that these compounds decreased acid production from glucose. More specifically, 68.3 µL and 104.7 µL of 0.3 N NaOH, respectively, were required to maintain a neutral pH in the presence of licoricidin and glabridin.

**Figure 5:**
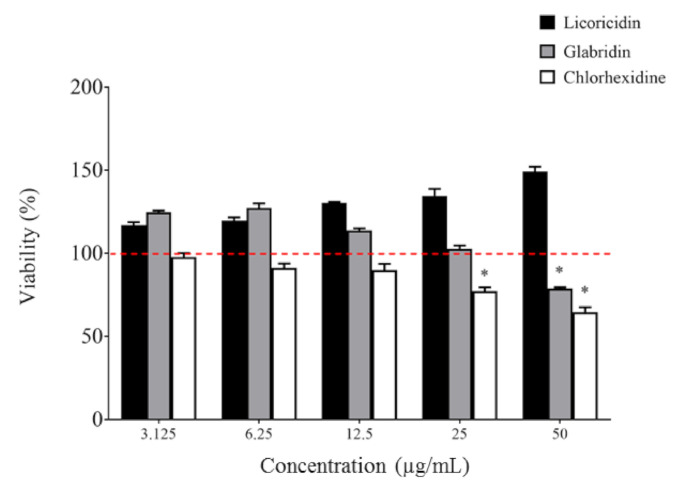
Effect of licoricidin and glabridin on the viability of oral keratinocytes. Cell viability was monitored following a 2-h exposure to the compounds using a MTT (3-[4,5-diethylthiazol-2-yl]-2,5-diphenyltetrazolium bromide) colorimetric assay. The assays were carried out in triplicate, and the means ± standard deviations were calculated. *, significantly different (*p* < 0.01) from the control (no compounds).

Biocompatibility is an important criterion when assessing novel molecules for oral clinical applications. The toxicity of licoricidin and glabridin was tested in an oral keratinocyte model and compared to chlorhexidine. As shown in Figure 5, licoricidin did not show any cytotoxicity against keratinocytes following a 2-h exposure, while glabridin, at the highest concentration tested (50 µg/mL), reduced the viability of keratinocytes by 20.9%. In comparison, chlorhexidine exhibited greater cytotoxicity. Interestingly, the percent cell viability increased for licoricidin (all concentrations tested) and for glabridin (≤12.5 µg/mL), suggesting that they have a positive effect on cell proliferation or metabolic activity.

In addition to the anti-caries properties identified in this study, licoricidin has been reported to possess anti-inflammatory activities that may be of interest for preventing inflammatory periodontal disease. More specifically, licoricidin inhibits the secretion of major cytokines and matrix metalloproteinases by lipopolysaccharide-stimulated human macrophages [25].

## 3. Materials and Methods

### 3.1. Compounds

Stock solutions (20 mg/mL) of glabridin (Wako Chemicals, Richmond, VA, USA) and licoricidin (EMMX Biotechnology, Lake Forest, CA, USA) were prepared in 95% ethanol and dimethyl sulfoxide, respectively. They were kept in the dark at 4 °C for up to one month. In all the assays described below, the corresponding concentrations of ethanol and dimethyl sulfoxide were used as controls and had no effect.

### 3.2. Bacteria and Growth Conditions

One reference strain (ATCC 25175) and four clinical strains (12A, 33A, INB, and T8) of *S. mutans* were used in this study. Unless indicated otherwise, the bacteria were grown aerobically at 37 °C in Brain Heart Infusion broth (BHI; BBL Microbiology Systems, Cockeysville, MD, USA) supplemented with 0.5% glucose.

### 3.3. Determination of Minimum Inhibitory and Minimum Bactericidal Concentrations

The minimum inhibitory concentrations (MIC) and minimum bactericidal concentrations (MBC) of licoricidin and glabridin were determined using a broth microdilution assay based on the National Committee for Clinical Laboratory Standards (NCCLS) [26]. Assays were performed in triplicate in three independent experiments, and a representative set of data is presented.

### 3.4. Biofilm Biomass and Viability

The ability of licoricidin and glabridin to eradicate *S. mutans* biofilms was investigated using a previously described protocol [27]. Briefly, the bacteria were grown in the wells of a 96-well microplate for 18 h, after which the spent medium and unattached bacteria were removed by aspiration. The biofilms were treated for 2 h at 37 °C with licoricidin or glabridin in 50 mM phosphate-buffered saline (PBS, pH 7.0) at their MIC and 2× MIC and were then washed once with distilled water. The biofilm biomass was quantified by staining with 0.01% crystal violet for 15 min. The wells were then washed twice with distilled water to remove unbound crystal violet and the plate was dried at 37 °C. One hundred µL of 75% (*v*/*v*) ethanol was added to each well, and the plate was shaken for 15 min to release the dye from the biofilms. Absorbance at 550 nm (A_550_) was measured using a Synergy 2 microplate reader (BioTek Instruments, Winooski, VT, USA). The ability of licoricidin and glabridin to cause the killing of *S. mutans* cells embedded in biofilms was also investigated using a previously described protocol with slight modifications [28]. Biofilms were formed in the wells of a 96-well, black-wall, clear-bottom microplate (Greiner Bio One, Frickenhausen, Germany) and treated with licoricidin and glabridin as described above. Bacterial viability was assessed using a Filmtracer™ Live/Dead Biofilm Viability kit (Thermo Fisher Scientific, Waltham, MA, USA) according to the manufacturer’s protocol. The assays were carried out in triplicate, and the means ± standard deviations were calculated.

### 3.5. Dextran Formation

*S. mutans* ATCC 25175 was grown for 18 h in BHI broth supplemented with 1% sucrose. The cultures were diluted in fresh medium to obtain an optical density at 660 nm (OD_660_) of 0.1. Equal volumes (50 µL) of bacterial culture, licoricidin or glabridin at 1/4 MIC, and Cascade Blue-conjugated dextran dye (0.3 mg/mL; Thermo Fisher Scientific) were added to the wells of a 96-well, black-wall, clear-bottom microplate. After an 18-h incubation at 37 °C, bacterial growth was assessed by recording the OD_660_, while dextran formation was quantified by determining the relative fluorescence units (RFU; excitation wavelength 495 nm; emission wavelength 525 nm) using a Synergy 2 microplate reader. The assays were carried out in triplicate and the means ± standard deviations were calculated

### 3.6. Adherence to Hydroxylapatite

To determine the effect of licoricidin and glabridin on the adherence of *S. mutans* ATCC 25175 to hydroxylapatite, bacteria were first labeled with fluorescein isothiocyanate (FITC) using a previously described protocol [29]. Hydroxylapatite-coated, 96-well, black-wall, clear-bottom microplates were prepared according to the procedure described by Shahzad et al. [30]. Parafilm-stimulated saliva was collected from six non-smoking healthy volunteers with the approval of the Université Laval Ethics Committee for Research in Humans (2015-237). The volunteers were asked to sign an informed consent form. Drinking and eating were not allowed for 2 h prior to collecting the saliva. The saliva samples (≈5 mL) were pooled, centrifuged (15,000× *g* for 10 min), and filter-sterilized. Saliva (100 µL) was added to each well, and the plate was incubated for 30 min at room temperature. The wells were then rinsed three times with PBS. FITC-labeled *S. mutans* (100 µL, OD_660_ = 0.5) cells were added to the wells together with either licoricidin or glabridin at concentrations ranging from 6.25 to 50 µg/mL. The plate was incubated for a further 2 h at 37 °C under low agitation in the dark. Unbound bacteria were removed by aspiration, and the wells were washed twice with PBS. RFUs (excitation wavelength 495 nm; emission wavelength 525 nm) corresponding to the level of bacterial adherence were determined using a Synergy 2 microplate reader. Control wells without test compounds were used to determine 100% adherence values while wells with no bacteria were used as controls to determine basal autofluorescence. The assays were carried out in triplicate, and the means ± standard deviations were calculated.

### 3.7. Glycolytic pH Drop Assay

The effect of licoricidin and glabridin on *S. mutans* glycolysis was assessed using a glycolytic pH drop assay. *S. mutans* ATCC 25175 cells from an 18-h culture were harvested by centrifugation and were suspended in a saline solution (50 mM potassium chloride + 1 mM magnesium chloride; pH 7.0) to an OD_660_ of 2. The bacteria were incubated in one of the following solutions: (i) saline, (ii) saline + 0.1% glucose, (iii) saline + 0.1% glucose + 6.25 µg/mL of licoricidin (1× MIC), or (iv) saline + 0.1% glucose + 25 µg/mL of glabridin (1× MIC). Glycolysis was monitored at a constant pH of 7.0 with a Radiometer PHM64 Standard pH meter and TTT_60_ (Radiometer, Copenhagen, Denmark) by automatic titration of the acids produced by the addition of 0.3 N NaOH over a 70-min period at 37 °C. The volume of 0.3 N NaOH added to maintain a constant pH of 7.0 was determined. Four independent experiments were performed and a representative set of data was presented.

### 3.8. In Vitro Biocompatibility

The human oral keratinocyte cell line B11 [31], which was kindly provided by S. Groeger (Justus Liebig University Giessen, Germany), was cultured in keratinocyte serum-free medium (K-SFM; Life Technologies Inc., Burlington, ON, Canada) supplemented with growth factors (50 µg/mL of bovine pituitary extract and 5 ng/mL of human epidermal growth factor) and 100 µg/mL of penicillin G-streptomycin. The cells were seeded (1 *×* 10^5^ cells in 100 µL) in the wells of a 96-well microplate (100 µL/well) and were cultured overnight at 37 °C in a 5% CO_2_ atmosphere until they reached confluence. The keratinocytes were treated for 2 h with either licoricidin, glabridin, or chlorhexidine (3.125 to 50 µg/mL). The wells were then immediately washed with DMEM prior to assessing cell viability using an MTT (3-[4,5-diethylthiazol-2-yl]-2,5-diphenyltetrazolium bromide) assay performed according to the manufacturer’s protocol (Roche Diagnostics, Mannheim, Germany). The assays were carried out in triplicate, and the means ± standard deviations were calculated.

### 3.9. Statistical Analysis

Statistical analysis was performed using a one-way analysis of variance with a post hoc Bonferroni multiple comparison test (GraphPad Software Inc.; La Jolla, CA, USA). Results were considered statistically significant at *p* < 0.01 or *p* < 0.001.

## 4. Conclusions

Compounds exhibiting dual antibacterial and anti-adherence effects may be promising therapeutic candidates for controlling biofilm-mediated diseases such as dental caries. In this study, we showed that licoricidin and glabridin exhibit antibacterial activity against biofilm-embedded and planktonic *S. mutans* cells. The two compounds also inhibited bacterial adherence to hydroxylapatite and acid production from glucose, and displayed low cytotoxicity for oral keratinocytes. Within the limitations of this study, including the use of a monospecies that does not mimic the complex oral microbial community, our results suggest that these licorice compounds could be added to oral hygiene products such as oral rinses, varnishes, and dentifrices in order to prevent dental caries.

## Figures and Tables

**Table 1:** Minimum inhibitory concentrations (MICs) and minimum bactericidal concentrations (MBCs) of licoricidin and glabridin against five strains of *Streptococcus mutans.* Assays were done in triplicate and a representative set of data is presented.

Compound	*S. mutans* Strains				
	25175	12A	33A	INB	T8
Licoricidin					
MIC (µg/mL)	6.25	6.25	6.25	6.25	6.25
MBC (µg/mL)	25	6.25	12.5	12.5	25
Glabridin					
MIC (µg/mL)	12.5	6.25	12.5	12.5	12.5
MBC (µg/mL)	25	6.25	12.5	25	25

**Table 2:** Effect of licoricidin and glabridin on the viability of preformed *Streptococcus mutans* biofilms following a 2-h treatment. The assays were carried out in triplicate, and the means ± standard deviations were calculated. *, significantly different (*p* < 0.001) from the control.

Strains	Biofilm Viability				
	Control	Licoricidin		Glabridin	
		MIC	2× MIC	MIC	2× MIC
**25175**	100 ± 0.4	98.1 ± 0.6	92.7 ± 2.1 *	93.7 ± 1.6 *	82.8 ± 1.3 *
12A	100 ± 1.6	100.2 ± 0.5	97.3 ± 1.1	100 ± 0.9	95.9 ± 1.4
33A	100 ± 1.5	91.5 ± 1.6 *	84.6 ± 1.9 *	82.7 ± 5.4 *	71.0 ± 2.8 *
INB	100 ± 2.1	96.1 ± 2.1	92.8 ± 2.2	92.4 ± 0.9	87.1 ± 1.8 *
T8	100 ± 2.0	99.6 ± 1.1	98.1 ± 1.3	94.9 ± 1.7	82.5 ± 0.8 *

## Data Availability

The data presented in this study are available on request from the corresponding author.
